# A Comparison of Anthropometric Measures for Assessing the Association between Body Size and Risk of Chronic Low Back Pain: The HUNT Study

**DOI:** 10.1371/journal.pone.0141268

**Published:** 2015-10-27

**Authors:** Ingrid Heuch, Ivar Heuch, Knut Hagen, John-Anker Zwart

**Affiliations:** 1 Department of Neurology and FORMI, Oslo University Hospital, Oslo, Norway; 2 Department of Mathematics, University of Bergen, Bergen, Norway; 3 Department of Neuroscience, Norwegian University of Science and Technology, Trondheim, Norway; 4 Norwegian National Headache Centre, Department of Neurology, St. Olavs Hospital, Trondheim, Norway; 5 Faculty of Medicine, University of Oslo, Oslo, Norway; McMaster University, CANADA

## Abstract

**Background:**

Previous work indicates that overweight and obese individuals carry an increased risk of experiencing chronic low back pain (LBP). It is not known, however, how the association with body size depends on the choice of anthropometric measure used.

**Objective:**

This work compares relationships with LBP for several measures of body size. Different results may indicate underlying mechanisms for the association between body size and risk of LBP.

**Methods:**

In a cohort study, baseline information was collected in the community-based HUNT2 (1995–1997) and HUNT3 (2006–2008) surveys in Norway. Participants were 10,059 women and 8725 men aged 30–69 years without LBP, and 3883 women and 2662 men with LBP at baseline. Associations with LBP at end of follow-up were assessed by generalized linear modeling, with adjustment for potential confounders.

**Results:**

Relationships between waist-hip-ratio and occurrence of LBP at end of follow-up were weak and non-significant after adjustment for age, education, work status, physical activity, smoking, lipid levels and blood pressure. Positive associations with LBP at end of follow-up were all significant for body weight, BMI, waist circumference and hip circumference after similar adjustment, both in women without and with LBP at baseline, and in men without LBP at baseline. After additional mutual adjustment for anthropometric measures, the magnitude of the association with body weight increased in women without LBP at baseline (RR: 1.130 per standard deviation, 95% CI: 0.995–1.284) and in men (RR: 1.124, 95% CI 0.976–1.294), with other measures showing weak associations only.

**Conclusion:**

Central adiposity is unlikely to play a major role in the etiology of LBP. Total fat mass may be one common factor underlying the associations observed. The association with body weight remaining after mutual adjustment may reflect mechanical or structural components behind the relationship between overweight or obesity and LBP.

## Introduction

Both obesity and low back pain (LBP) represent major health problems in today's society. Obesity is associated with serious disorders as cardiovascular disease, diabetes and cancer [1]. LBP causes more global disability than any other condition [2], with immense costs to society in terms of suffering and lost productivity [3]. It is therefore important to determine risk factors for LBP and explore potential relations with obesity.

The great majority of the cases of LBP seen in clinical practice are nonspecific and cannot be assigned any particular pathoanatomical diagnosis [4]. Yet at the population level, epidemiologic studies have shown associations between occurrence of LBP and risk factors such as physical activity [5], work status [6] and cigarette smoking [7], although results from different populations have not always been consistent. It has also been suspected for a long time that overweight and obesity may be associated with the risk of LBP [8], but despite the large number of studies exploring this relationship, early reviews [9–11] did not reach any firm conclusion. The main impression was that extreme obesity was likely to confer a higher risk of LBP but that definite statements could not be made about overweight. A more recent meta-analysis [12] summarizing results reported until 2009 concluded, however, that the overall evidence, incorporating results from cross-sectional and prospective studies, supported an increased risk of LBP for both overweight and obesity. This result was confirmed in additional large prospective studies [13,14] carried out after the meta-analysis [12].

Most population studies of associations with LBP have focused on body mass index (BMI) as the measure of body size, with overweight and obesity defined in terms of BMI. Some studies have also dealt with associations with body weight [15–17]. Waist circumference has been considered as an alternative measure in quite a few studies [18–20], sometimes in combination with the waist-hip-ratio [17,21,22]. The total evidence supporting an association between body size and risk of LBP still rests essentially on data involving BMI.

Various mechanisms have been proposed to explain associations between body size and LBP [12,23]. A heavy mechanical load may lead to greater compressive forces or increased shear on the structures of the lumbar spine [12]. Structural modifications involving disc degeneration [24] or Modic changes in vertebral endplates [25] may be related to increasing loads. Moreover, impaired spinal mobility in overweight individuals [26] may affect disc nutrition [12]. Blood supply to the lumbar region may also be affected by atherosclerosis [27], which in turn may be associated with adiposity. More generally, a large amount of fat tissue may lead to an elevated production of cytokines and acute-phase reactants [28], activating pro-inflammatory pathways and producing pain. Finally, emotional [9] or behavioral [23] components may play a certain role.

A comparison of associations between various measures of body size and LBP may provide a clue as to which underlying mechanisms are most plausible. BMI constitutes the classic standardized measure of body size which is closely related to overall subcuteaneous fat mass [29] but also represents skeletal muscle mass, especially in men [30]. BMI does not, however, reflect the distribution of the fat mass, in contrast to a measure such as waist circumference, which is more strongly correlated with abdominal fat mass, both subcutaneous and intraabdominal [29]. The waist-hip-ratio often seems to serve as a better standardized measure of central adiposity than BMI or waist circumference, in particular among elderly [31]. Hip circumference regarded as a separate measure of body size may also be associated with health indicators in different ways from waist circumference and BMI [32], perhaps reflecting other aspects of the distribution of fat and muscle tissue. Simple body weight is still strongly related to fat mass percentage [33].

If mechanical or structural factors play a major role in explaining associations between body size and LBP, strong associations should be observed in particular considering body weight and maybe BMI, although other measures could also be affected. If, on the other hand, physiological mechanisms are important, especially those involving abdominal fat mass and inflammation, measures such as waist circumference or waist-hip-ratio should show strong associations. To distinguish between such possibilities, it is essential to study associations with different relevant measures in the same data set, with standardized adjustment for other risk factors. Although relevant causal mechanisms may be quite different, similar comparisons of associations with different measures of body size have been performed for risk of cardiovascular disease and cancer and for overall mortality. Some studies have indicated that measures of abdominal obesity may have a predictive ability which is at least equal to that of BMI [34–36].

In this study, which is based on an 11 year follow-up of a large Norwegian cohort of adults, we compare associations with occurrence of chronic LBP for body weight, BMI, waist circumference, hip circumference and waist-hip-ratio. Separate analyses are performed to study risk of subsequent LBP among subjects without LBP at baseline and recurrence or persistence of LBP among those reporting LBP at baseline. The same data set has previously been used to study associations between occurrence of LBP and body height [37] and overweight and obesity as assessed by BMI [14].

## Material and Methods

### Study design

The Nord-Trøndelag Health Study (HUNT) is a population-based study of an entire county in Norway, with information on health status being collected over a long period [38]. Three consecutive surveys have been carried out; HUNT1 in 1984–86 [39], HUNT2 in 1995–97 [38] and HUNT3 in 2006–08 [40]. The work presented here is based on data from the HUNT2 and HUNT3 surveys.

In 1995–97 all residents of Nord-Trøndelag aged 20 years or more received an invitation to participate in the HUNT2 survey, with a questionnaire enclosed on health status [38]. One question was formulated in this manner: "During the last year, have you had pain and/or stiffness in your muscles and limbs that has lasted for at least 3 consecutive months?" If a respondent answered in the affirmative, the following question was asked: "Where did you have pain and/or stiffness?" The lower back was one site listed among several possibilities. Respondents checking this alternative were regarded as suffering from chronic LBP [41]. Participants were also invited to a clinical consultation which included measurement of height, weight and waist and hip circumference. Additional measurements were made of blood pressure and lipid levels in non-fasting blood. Among a total population of 30,022 men and 28,906 women in the age interval 30–69 years, 21,442 men and 23,205 women indicated whether they suffered from chronic LBP and had measurements performed of height, weight, waist and hip, corresponding to a participation rate of 75.8%.

Similar information was collected in the HUNT3 survey, carried out in 2006–2008 with a corresponding target population. Only information on status of back pain from HUNT3 is considered in the present prospective study, in combination with the baseline information from HUNT2. The follow-up aimed at the cohort consisting of 44,647 individuals aged 30–69 years when they participated in the HUNT2 survey, with information available on height, weight, waist and hip circumference and presence or absence of chronic low back pain. Participants outside this age range in HUNT2 were excluded because of relatively low participation rates in the subsequent HUNT3 study [42]. During the period of follow-up, from HUNT2 to HUNT3, 2631 individuals in this cohort died, 1675 individuals left the county of Nord-Trøndelag, and one individual disappeared. Furthermore, 15,012 members of the cohort residing in the county at the time of HUNT3 did not participate or did not supply information about low back pain. Thus a total of 25,329 participants, 11,387 men and 13,942 women, were available for analysis after follow-up, representing 62.8% of the remaining individuals resident in the county and 56.7% of the original cohort.

Each participant in the HUNT2 and HUNT3 surveys signed a written informed consent regarding the collection and use of data for research purposes. The work was approved by the Regional Committee for Medical and Health Research Ethics in Central Norway, and HUNT was also approved by the Norwegian Data Inspectorate.

### Exposure and covariate assessment

Height and weight were measured with the participants wearing light clothes without shoes, height to the nearest 1.0 cm and weight to the nearest 0.5 kg. Waist and hip circumferences were measured with a steel band to the nearest 1.0 cm, with participants standing and arms hanging relaxed. Waist circumference was measured horizontally at the height of the umbilicus, and hip circumference was measured at the thickest part of the hip. Body mass index was defined as weight/height^2^.

Baseline age was categorized into 10 year intervals. Education was grouped according to duration, as ≤9, 10–12, or ≥13 years. Categories of cigarette smoking represented current daily smoking, previous daily smoking, and never daily smoking.

For physical activity in leisure time, including going to work, one category included those engaged in light activity only or hard physical activity (leading to sweating or being out of breath) <1 hour per week. Other categories represented hard physical activity 1–2 and ≥3 hours per week.

Four categories of work status were defined, the first comprising people being employed or carrying out professional work. The second category included those temporarily out of work, students and individuals in military service. The third category included pensioners and people receiving social security support, and the fourth category represented women occupied full-time with housework. Those currently working supplied information about physical activity at work, in four categories representing substantially sedentary work, work involving extensive walking, work leading to both walking and lifting, and work involving particularly strenuous activities.

Systolic blood pressure was categorized as <120, 120–139, 140–159, ≥ 160 mmHg, and diastolic blood pressure as <80, 80–89, 90–99, ≥100 mmHg. Lipid levels in non-fasting blood were measured by enzymatic colorimetric procedures and were categorized by quintiles [43].

### Statistical analysis

The distributions of measures of body size were described separately for women and men by mean values and standard deviations within categories of LBP status at baseline. Interrelations between different measures were characterized by correlation coefficients.

Analyses of associations between anthropometric measures and risk and recurrence or persistence of chronic LBP at end of follow-up were conducted by generalized linear modeling with a log link, with adjustment for potential confounders. All analyses were carried out separately for women and men without and with LBP at baseline. One set of analyses included linear effects of sex-specific standardized versions of the measures of body size, producing relative risks of LBP per standard deviation of each measure. This procedure made it possible to compare the magnitude of the different associations even though the measured values were expressed in very different units. Linearity was checked for by adding quadratic and cubic terms to the regression models. Categorical analyses were based on a subdivision of the range for each measure in sex-specific quintiles, determined considering the total cohort at baseline. This approach also facilitated a direct comparison of the magnitude of associations for different measures.

Adjustment was first made for age only in a basic set of analyses. Additional categorical adjustment was then performed for a number of factors likely to be associated both with the anthropometric measures and occurrence of LBP, such as education, work status, physical activity in leisure time and at work, smoking, lipid levels and blood pressure. Finally, mutual adjustment was also carried out among the anthropometric measures showing a significant influence in the separate analyses, considering linear effects.

As information was missing on some potential confounders in a minor fraction of the data set, the analyses with complete adjustment were based on a somewhat lower number of individuals than the basic age-adjusted analyses.

All statistical analyses were carried out using IBM SPSS version 21 (IBM Corp., Armonk, New York).

## Results

### Descriptive baseline measures

Mean values of the anthropometric measures were in general slightly greater among individuals with chronic LBP at baseline than in those without LBP, both in women and men (Table 1). Taking the variation in the population into account, expressed by the standard deviations (Table 1), the groups with and without LBP at baseline were still largely comparable.

**Table 1:** Mean values (with standard deviations) of anthropometric measures at baseline.

	Women				Men			
	Without LBP at baseline		With LBP at baseline		Without LBP at baseline		With LBP at baseline	
*n*	10,059		3883		8725		2662	
Body weight (kg)	70.2	(11.7)	72.3	(12.4)	84.3	(11.5)	84.6	(11.5)
BMI (kg/m^2^)	25.8	(4.2)	26.6	(4.4)	26.5	(3.2)	26.7	(3.3)
Waist circumference (cm)	80.0	(10.4)	82.4	(11.0)	91.6	(8.4)	92.5	(8.6)
Hip circumference (cm)	101.1	(8.8)	102.8	(9.0)	102.4	(5.7)	102.7	(5.7)
Waist-hip-ratio	0.790	(0.056)	0.800	(0.059)	0.894	(0.052)	0.900	(0.054)

LBP, low back pain; BMI, body mass index.

Correlations between anthropometric measures among participants without LBP at baseline (Table 2) were quite high, in the range from 0.76 to 0.91, except for the waist-hip-ratio which showed much lower correlations with body weight, BMI and hip circumference. No major differences were observed between women and men, although correlations in the upper range were slightly lower in men. Corresponding correlations computed among individuals with LBP at baseline were very similar to those displayed in Table 2.

**Table 2:** Correlations between anthropometric measures, among individuals without chronic LBP at baseline.

	Women					Men				
	Body weight	BMI	Waist circumference	Hip circumference	Waist-hip-ratio	Body weight	BMI	Waist circumference	Hip circumference	Waist-hip-ratio
Body weight	1.00	0.91	0.85	0.88	0.45	1.00	0.85	0.82	0.85	0.45
BMI		1.00	0.86	0.87	0.49		1.00	0.82	0.76	0.54
Waist circumference			1.00	0.85	0.75			1.00	0.78	0.80
Hip circumference				1.00	0.30				1.00	0.26
Waist-hip-ratio					1.00					1.00

BMI, body mass index.

### Associations with risk of LBP

With adjustment for age only, all measures of body size showed highly significant positive relationships with risk of LBP among individuals not affected by LBP at baseline, in women as well as in men (Table 3). In women, the strongest association was seen for waist circumference, although the strength of the association was quite similar for body weight and BMI. In men, BMI showed the strongest association with risk of LBP, followed by body weight and waist circumference.

**Table 3:** Log-linear associations between anthropometric measures and risk of chronic LBP, among individuals without chronic LBP at baseline.

	Women			Men		
	Adjustment for age only	Complete adjustment for other risk factors^a^	Additional mutual adjustment^b^	Adjustment for age only	Complete adjustment for other risk factors^a^	Additional mutual adjustment^b^
	Risk ratio^c^ (95% CI)	Risk ratio^c^ (95% CI)	Risk ratio^c^ (95% CI*)*	Risk ratio^c^ (95% CI)	Risk ratio^c^ (95% CI)	Risk ratio^c^ (95% CI)
Body weight	1.092 (1.049–1.136)	1.087 (1.039–1.138)	1.130 (0.995–1.284)	1.115 (1.059–1.174)	1.091 (1.030–1.157)	1.124 (0.976–1.294)
*P*	< 0.001	< 0.001	0.06	< 0.001	0.003	0.11
BMI	1.092 (1.049–1.137)	1.075 (1.023–1.128)	0.956 (0.846–1.080)	1.125 (1.068–1.184)	1.091 (1.027–1.158)	1.062 (0.944–1.196)
*P*	< 0.001	0.004	0.47	< 0.001	0.004	0.32
Waist circumference	1.097 (1.053–1.143)	1.078 (1.025–1.134)	1.006 (0.908–1.115)	1.108 (1.051–1.169)	1.064 (1.001–1.131)	0.951 (0.844–1.072)
*P*	< 0.001	0.004	0.91	< 0.001	0.05	0.41
Hip circumference	1.078 (1.035–1.123)	1.073 (1.024–1.123)	0.995 (0.897–1.104)	1.085 (1.029–1.144)	1.060 (1.000–1.123)	0.955 (0.853–1.068)
*P*	< 0.001	0.003	0.92	0.003	0.05	0.42
Waist-hip ratio	1.073 (1.032–1.116)	1.033 (0.985–1.084)		1.090 (1.031–1.151)	1.037 (0.975–1.102)	
*P*	< 0.001	0.18		0.002	0.25	

LBP, low back pain; CI, confidence interval; BMI, body mass index.

^a^Adjustment for age, education, work status, physical activity, smoking, HDL-cholesterol, triglycerides and blood pressure.

^b^Additional mutual adjustment for body weight, BMI, waist circumference and hip circumference.

^c^Per standard deviation of each variable.

After additional adjustment for education, work status, physical activity, smoking, HDL-cholesterol, triglycerides and blood pressure, the association with waist-hip-ratio became much weaker and was no longer significant, neither in women nor in men (Table 3). The strength of the association was reduced to some extent for body weight, BMI and waist and hip circumference, but all associations remained highly significant in women, although the significance for waist and hip circumference was marginal in men. Considering categorical risk estimates for body weight, BMI and waist and hip circumference with similar adjustment, the risk generally increased with increasing quintiles of each anthropometric measure (Table 4), with some exceptions, especially in men. Tests for additional quadratic and cubic terms did not show any significant deviation from the log-linear relationships reported in Table 3, and results based on these relationships generally agreed with those from the categorical analysis. For example, the risk ratio of 1.087 per standard deviation for body weight (11.7 kg), shown for women in Table 3, corresponds to a risk ratio of 1.087^2^ = 1.182 for a comparison between individuals 2 standard deviations (23.4 kg) apart and a risk ratio 1.087^3^ = 1.284 for individuals 3 standard deviations (35.1 kg) apart.

**Table 4:** Risk of chronic LBP by quintiles of body weight, BMI and waist and hip circumference, among individuals without chronic LBP at baseline.

	Women			Men		
	Quintile range	Risk ratio^a^ (95% CI)		Quintile range	Risk ratio^a^ (95% CI)	
Body weight (kg)						
	≤ 61.0	1.00	(reference)	≤ 74.5	1.00	(reference)
	61.5–66.5	1.03	(0.90–1.18)	75.0–80.5	1.24	(1.04–1.48)
	67.0–72.5	1.17	(1.02–1.33)	81.0–86.0	0.97	(0.80–1.18)
	73.0–80.0	1.17	(1.02–1.34)	86.5–93.5	1.17	(0.97–1.41)
	≥ 80.5	1.23	(1.06–1.42)	≥ 94.0	1.36	(1.13–1.64)
BMI (kg/m^2^)						
	≤ 22.6	1.00	(reference)	≤ 23.9	1.00	(reference)
	22.7–24.5	1.03	(0.90–1.17)	24.0–25.6	1.01	(0.84–1.21)
	24.6–26.6	1.19	(1.04–1.35)	25.7–27.2	1.06	(0.88–1.26)
	26.7–29.5	1.15	(1.01–1.33)	27.3–29.2	1.14	(0.95–1.37)
	≥ 29.6	1.27	(1.09–1.47)	≥ 29.3	1.31	(1.08–1.58)
Waist circumference (cm)						
	≤ 72	1.00	(reference)	≤ 85	1.00	(reference)
	73–77	1.15	(1.00–1.31)	86–90	0.90	(0.75–1.08)
	78–82	1.23	(1.08–1.40)	91–94	1.03	(0.86–1.23)
	83–90	1.21	(1.05–1.40)	95–99	1.09	(0.91–1.32)
	≥ 91	1.28	(1.10–1.49)	≥ 100	1.07	(0.88–1.31)
Hip circumference (cm)						
	≤ 94	1.00	(reference)	≤ 98	1.00	(reference)
	95–99	0.98	(0.86–1.12)	99–101	1.06	(0.89–1.27)
	100–103	1.16	(1.02–1.32)	102–104	1.13	(0.95–1.35)
	104–109	1.20	(1.05–1.37)	105–107	1.07	(0.90–1.28)
	≥ 110	1.15	(1.00–1.33)	≥ 108	1.22	(1.02–1.47)

LBP, low back pain; BMI, body mass index; CI, confidence interval.

^a^Adjustment for age, education, work status, physical activity, smoking, HDL-cholesterol, triglycerides and blood pressure.

As the waist-hip-ratio did not exhibit major associations with risk of LBP after adjustment for potential confounders, this factor was dropped in further analyses carried out with mutual adjustment for the anthropometric factors considered (Table 3). Both in women and men, the estimated association with body weight was strengthened in this analysis, although formal statistical significance was no longer attained. Among men, BMI also retained a moderate positive association with LBP in this analysis, but otherwise the risk ratios did no longer reflect apparent associations. Separate analyses, not included in Table 3, showed that the association with BMI among women disappeared already after adjustment for body weight. In contrast, adjustment for waist or hip circumference led to a marked reduction but not complete elimination of the association with BMI. On the other hand, the association with body weight was strengthened by adjustment for BMI, and to some extent also by separate adjustment for waist or hip circumference. The associations with waist or hip circumference were substantially reduced by adjustment for BMI and completely disappeared after adjustment for body weight. A similar pattern was seen in men after partial mutual adjustment, except for the effect of BMI which was not entirely eliminated.

Particular analyses were also carried out to assess associations with body weight with additional adjustment for body height but not BMI. The estimated standardized associations with body weight were in these situations very similar to those shown for corresponding analyses of BMI, not including body weight or height.

### Associations with recurrence or persistence of LBP

In age-adjusted analyses restricted to women who reported LBP at baseline, all measures of body size showed significant positive associations with occurrence of LBP at follow-up (Table 5). Similar significant associations were only seen for BMI and waist circumference in men, with no significant associations remaining after adjustment for other potential confounders. Among women, the association with waist-hip-ratio was no longer significant after such adjustment. No definite associations were observed in either sex after further mutual adjustment among the anthropometric measures (Table 5).

**Table 5:** Log-linear associations between anthropometric measures and recurrence or persistence of chronic LBP, among individuals with chronic LBP at baseline.

	Women			Men		
	Adjustment for age only	Complete adjustment for other risk factors^a^	Additional mutual adjustment^b^	Adjustment for age only	Complete adjustment for other risk factors^a^	Additional mutual adjustment^b^
	Risk ratio^c^ (95% CI)	Risk ratio^c^ (95% CI)	Risk ratio^c^ (95% CI)	Risk ratio^c^ (95% CI)	Risk ratio^c^ (95% CI)	Risk ratio^c^ (95% CI)
Body weight	1.073 (1.048–1.098)	1.065 (1.037–1.094)	1.030 (0.951–1.116)	1.015 (0.973–1.059)	1.005 (0.960–1.052)	0.906 (0.814–1.009)
*P*	< 0.001	< 0.001	0.46	0.49	0.84	0.07
BMI	1.067 (1.043–1.092)	1.060 (1.032–1.088)	0.992 (0.922–1.068)	1.044 (1.002–1.088)	1.032 (0.986–1.079)	1.090 (0.997–1.192)
*P*	< 0.001	< 0.001	0.84	0.04	0.18	0.06
Waist circumference	1.071 (1.046–1.096)	1.066 (1.035–1.099)	1.006 (0.944–1.072)	1.044 (1.002–1.087)	1.019 (0.974–1.067)	1.017 (0.937–1.103)
*P*	< 0.001	< 0.001	0.86	0.04	0.41	0.70
Hip circumference	1.074 (1.049–1.099)	1.069 (1.040–1.098)	1.042 (0.978–1.111)	1.032 (0.989–1.076)	1.016 (0.971–1.062)	1.023 (0.938–1.115)
*P*	< 0.001	< 0.001	0.20	0.15	0.49	0.61
Waist-hip ratio	1.038 (1.011–1.065)	1.021 (0.989–1.054)		1.038 (0.997–1.081)	1.014 (0.969–1.061)	
*P*	0.005	0.20		0.07	0.55	

LBP, low back pain; CI, confidence interval; BMI, body mass index.

^a^Adjustment for age, education, work status, physical activity, smoking, HDL-cholesterol, triglycerides and blood pressure.

^b^Additional mutual adjustment for body weight, BMI, waist circumference and hip circumference.

^c^Per standard deviation of each variable.

## Discussion

This work attempts to compare associations with chronic LBP for several measures of body size, despite strong correlations between the measures considered. After adjustment for other risk factors, the four variables body weight, BMI, waist circumference and hip circumference all showed about equally strong associations with LBP in women free of LBP at baseline. In men, body weight and BMI appeared to be somewhat stronger predictors than waist and hip circumference. Mutual adjustment for anthropometric measures produced a different overall impression, and only body weight seemed to incorporate a component with an independent effect on risk of LBP among women. In men, BMI still appeared to play a certain role in addition to body weight, after effects of the other measures on risk had been taken into account. In contrast, for participants who reported LBP at baseline, no particular measure seemed to have an independent effect on the probability of having LBP at end of follow-up after mutual adjustment, except perhaps for BMI in men.

Our results are based on a relatively large population-based data set with standardized anthropometric measurements performed at baseline in connection with a clinical examination. The intervening period before final assessment of LBP was long enough for possible effects related to anthropometric features to become manifest. Baseline information was available on potential confounders and it was possible to carry out separate analyses among those free of LBP at baseline and those affected. Unfortunately no information was collected about LBP at other times. Moreover, the LBP status recorded at baseline and end of follow-up relied on information provided by the participants, and no attempt was made to characterize the severity of the pain. Although the initial survey at baseline had a reasonable response rate, participation was unfortunately lower in the final survey and may have depended to some extent on socioeconomic status [42].

Associations with BMI among adults have been examined in a great many studies of LBP, but few studies have dealt with body weight as a separate risk factor, probably because of the strong correlation between the two variables. In some prospective studies broadly similar results have been found for the two variables, often with definite positive associations with both body weight and BMI in women and less definite results in men [15,16]. A cross-sectional study found no clear associations at all [17]. Results regarding waist circumference have been more varied. Some cross-sectional studies [21,22] have shown positive associations between waist circumference and prevalence of LBP in women, in parallel with similar associations with BMI, but no associations among men. In one prospective study [20] including both sexes, waist circumference showed a positive association with occurrence of radiating LBP but not with non-specific LBP. A marginal positive association was found in a short-term prospective study of male conscripts [19]. Hip circumference showed a positive relationship with prevalence of LBP among women in one study [22] but not in another [17]. Waist-hip-ratio was associated with occurrence of LBP in women in one prospective study [44] and a couple of cross-sectional studies [21,22,45], with no relationship being found among men. A small case-control study [17] reported an inverse association with waist-hip-ratio.

As estimates have been computed with different measurement units or categorization and with different adjustment, it is difficult to compare the strength of the associations for the various anthropometric factors considering results from separate studies. In one relatively large cross-sectional study [21], waist circumference clearly exhibited a stronger association with LBP among women than did BMI or waist-hip-ratio. In an analysis of associations with BMI and waist and hip circumference among young adults [22], the positive association with waist circumference was the only one remaining after complete mutual adjustment.

Both our analyses of risk of LBP among individuals without LBP at baseline and our analyses of recurrence or persistence indicate that the age-adjusted association seen with the waist-hip-ratio largely disappears after adjustment for other risk factors. This suggests that central adiposity in itself is not an important factor for explaining the occurrence of LBP. This result does not carry similar implications about total fat mass, as the waist-hip-ratio shows much weaker correlations with total fat mass than the other measures considered [46].

In women without LBP at baseline, the variables body weight, BMI, waist and hip circumference all showed significant positive associations with occurrence of LBP at end of follow-up. All four associations may to a large extent reflect a common underlying relationship between total fat mass and LBP. If this hypothesis is correct, any association remaining after mutual adjustment should mainly represent other underlying explanatory components. Actually body weight was the only relevant predictor of risk after mutual adjustment. This may indicate that heavy mechanical or structural loads on the spine play an essential role in the etiology of LBP among women. In men without LBP at baseline, a somewhat different picture emerged, with body weight and BMI showing stronger associations than waist and hip circumference after adjustment for other risk factors. Moreover, the association with BMI did not entirely disappear after mutual adjustment. The overall interpretation is still similar to that in women, with results being consistent with a common relationship between total fat mass and LBP, in addition to a strong mechanical or structural component. The minor discrepancies observed between results in women and men may reflect general sex differences in body structure. The lack of association seen among individuals with LBP at baseline after mutual adjustment suggests that mechanical or structural factors may not be equally important for persistence or recurrence of LBP, although a common underlying relationship mediated by total fat mass may still play a major role, in particular in women.

It should be emphasized that waist and hip circumference may in general still be essential predictors of risk of LBP, despite the finding that these covariates did not contribute to prediction after mutual adjustment for body measures. The mutually adjusted analyses are only meant to reveal additional associations specific to particular measures, above potential associations reflecting common underlying factors. The major correlations between anthropometric measures displayed in Table 2 will greatly influence any regression analysis attempting to separate independent effects in this manner. For ordinary interpretation of relationships between each measure and risk of LBP, the separate risk ratios shown in the middle columns for each sex in Tables 3 and 5, or the categorical estimates in Table 4, describe the relevant associations.

Our attempt to compare associations with LBP for different measures of body size is similar to attempts made for other medical conditions. Comparisons have frequently been carried out by examining the magnitude of the separate associations after adjustment for other potential confounders, as for risk of cardiovascular disease [47], for risk of incident diabetes [48] or for overall or cause specific mortality [35,49]. Measures such as waist circumference [35] or waist-hip-ratio [49] have in many cases shown stronger associations with mortality than BMI. In some studies mutual adjustment has also been carried out to determine whether particular risk factors confer an additional risk above that predicted by other measures. This has mostly been achieved by categorical adjustment, but there are also examples of continuous mutual adjustment [50]. It has been pointed out that categorical modeling may not be sufficient in these situations [51] when the strong correlation between predictors is taken into account.

Few studies of LBP have been carried out considering the body distribution of different tissues. In one study [45], central obesity and loss of muscle mass in the trunk and lower extremities were associated with the occurrence of non-sciatic LBP in women. In another study [52] higher levels of LBP intensity and disability were associated with body fat mass but not lean tissue mass.

The idea that the total fat mass may influence the risk of LBP is consistent with our results before mutual adjustment, but other more specific explanations are needed to account for the results after such adjustment. Structural degenerative changes may be responsible for relationships between body size and LBP [12], although it is not clear whether degenerative changes as seen on MRI are actually related to LBP [53]. Thus disc degeneration seems to be related to overweight [24,54] and a reduced disc height has been observed among obese [55]. Bariatric surgery, with subsequent weight reduction, has also been associated with increased intervertebral disc height [56]. Moreover, BMI and waist circumference have been found to be related to vertebral endplate changes [25]. It is more difficult to use our results to evaluate theories about associations between body size and LBP involving emotional or behavioral components or impaired blood supply to the lumbar region. It does not seem reasonable, however, that such theories should favor a relationship operating through body weight in particular.

This study apparently constitutes the first attempt in a cohort setting to compare associations with risk of LBP systematically for different measures of body size. Body height has already been found to be associated with risk of LBP among women in this data set [37]. For cardiovascular disease corresponding results may be important in the development of simple procedures to predict the risk pertaining to any particular individual, but this problem hardly arises for LBP. Our results may still contribute to the understanding of causal mechanisms, which in turn will make it easier to formulate suitable recommendations for prevention of LBP. At the moment it is difficult to provide comprehensive advice of this kind. Future work on risk of LBP should preferably take into account more detailed information about the relative amount of different body tissues, although it is currently expensive to obtain such information in large populations.

In connection with rehabilitation of LBP patients it has been stated that BMI may not be the most suitable measure of obesity to predict the outcome [57]. The data presented here indicate that simple body weight may provide at least an equally good basis for describing the risk of LBP as BMI, although more precise estimates of body composition may still offer considerably better possibilities.
